# Viral load testing coverage and suppression rate among children and adolescents on ART in Sierra Leone

**DOI:** 10.5588/pha.25.0053

**Published:** 2026-03-06

**Authors:** F. Kanu, F. Lansana, M. Mustapha, E.K. Tabor, P. Thekkur, I.F. Kamara, B.D. Fofanah, J.S. Kanu, N. Sesay, I.S. Turay, J.A. Koroma, W.K. Lahai, A.M. Falama, M.A. Sesay, S. Lakoh

**Affiliations:** 1 National AIDS Control Program, Ministry of Health, Freetown, Sierra Leone;; 2 College of Medicine and Allied Health Sciences, University of Sierra Leone, Freetown, Sierra Leone;; 3 World Health Organization Sierra Leone, Freetown, Sierra Leone;; 4 Centre for Operational Research, International Union Against Tuberculosis and Lung Disease, Pari, France;; 5 National Public Health Agency, Ministry of Health Sierra Leone, Freetown, Sierra Leone;; 6 Ola During Children’s Hospital, Ministry of Health, Freetown, Sierra Leone;; 7 Directorate of Policy, Planning, and Information, Ministry of Health, Freetown, Sierra Leone;; 8 National TB Control Program, Ministry of Health, Freetown, Sierra Leone;; 9 National Malaria Control Program, Ministry of Health, Freetown, Sierra Leone;; 10 Directorate of Disease Prevention and Control, Ministry of Health, Freetown, Sierra Leone.

**Keywords:** PLHIV, children, adolescents, UNAIDS target, Sustainable Development Goals, SORT IT, operational research

## Abstract

**SETTING:**

Anti-retroviral treatment (ART) centres from eight districts of Sierra Leone.

**OBJECTIVES:**

Among children and adolescents (0–19 years) registered for ART after January 2023 and currently on care, to assess i) viral load (VL) testing coverage and viral suppression, ii) turnaround time from sample collection to results, and iii) factors associated with not undergoing testing.

**DESIGN:**

A cohort study using programmatic data extracted from April to October 2025.

**RESULTS:**

Of 950 participants, 931 (98%) were eligible for VL testing (on ART for ≥6 months). Of the total participants, 286 (30%) underwent VL testing at least once (coverage), of which 247 (86%) had viral suppression. Median turnaround time was 36 days, ranging from 6 days in districts with centralised VL testing laboratory to 65 days in a district far from the central laboratory. Participants from ART centres situated in the Western Area Urban district (adjusted relative risk: 1.8, 95% confidence interval: 1.0–3.1) were at higher risk of not undergoing VL testing.

**CONCLUSION:**

Although high viral suppression was appreciable, less than one third of children and adolescents underwent VL testing with a long turnaround time, delaying virological failure detection and appropriate care. The national AIDS control programme should decentralise VL testing and strengthen sample transportation to ensure equitable access.

Globally, an estimated 40.8 million people were living with HIV in 2024, of which about 2.4 million were children and adolescents (aged 0–19 years).^1^ Despite a decline in HIV incidence among children (62%) and adolescents (44%) since 2010, an estimated 2,70,000 were newly infected in 2024, with 86% of them from the African region alone.^1,2^ Additionally, of the 90,000 children and adolescents who died due to HIV-related causes globally, 75% were from the African region.^1,2^ In 2020, to meet the Sustainable Development Goal (SDG) target of ending the HIV epidemic by 2030, the UNAIDS set an ambitious target of achieving 95-95-95 by 2025; 95% of all people living with HIV should be diagnosed, 95% of those diagnosed should be on anti-retroviral therapy (ART), and 95% of those on ART should have viral suppression (<1,000 copies/mL).^3,4^ However, by 2025, the achievement was 87-89-95, with disproportionately lower achievement of 63-87-85 among children.^1^

Routine viral load (VL) testing is essential for monitoring viral suppression to detect treatment failure for switching to a second-line ART regimen and assess 95-95-95 metrics.^5^ Thus, the World Health Organization (WHO) recommends VL testing of all people living with HIV at 6 and 12 months after ART initiation and annually thereafter.^5^ However, a recent study from 12 African countries reported suboptimal VL testing coverage among children (68%) and adolescents (77%) receiving ART.^6^ The reasons for low VL testing coverage includes caregivers not bringing children to ART centres, difficulties with sample collection, and suboptimal laboratory capacity.^7,8^ A study from 31 countries reported a lower viral suppression rate among children and adolescents (64%) compared to adults (79%) with HIV at 1 year after ART.^9^ The reasons for low VL suppression include advanced disease stage at ART initiation, poor adherence, frequent drug stock-outs, lack of caregiver support, and suboptimal ART regimens.^10^ However, with the introduction of dolutegravir (DTG)-based ART regimens, the viral suppression rates are expected to improve among children and adolescents with HIV.^11^

Sierra Leone is a West African country with a HIV prevalence of 1.7% among individuals aged 15–49 years.^1,12^ In 2024, about 81,000 people were living with HIV in the country, of which about 10,500 were children and adolescents.^12^ The National AIDS Control Programme (NACP) under the Ministry of Health has committed to achieve 98-98-98 targets to end the AIDS epidemic by 2030.^13^ Despite the efforts of NACP, the progress towards achieving targets is slow, especially among children and adolescents.^14,15^ Sierra Leone reported to UNAIDS that about 87% of adults and 39% of children are tested and treated for HIV. Viral suppression rates are not routinely reported in Sierra Leone.^12,13^ However, a country-wide study reported the viral suppression rate of 88%, but without disaggregated information on children and adolescents with HIV.^11^

The NACP in partnership with the US Centres for Disease Control and Prevention enhanced the capacity of the two molecular laboratories to process 100,000 VL samples annually and trained ART staff in sample collection and transportation.^16^ Although 6-monthly VL testing was made part of the minimum care package for children and adolescents with HIV in January 2023, no systematic assessment of VL testing coverage and viral suppression was conducted to aid evidence-informed decisions for improving HIV care provision.^13^ Thus, a study was conducted in eight high-HIV-burden districts of Sierra Leone among children and adolescents who were initiated on ART after January 2023, to assess i) the proportion who have undergone VL testing and factors associated with not undergoing at least one VL test since registration, ii) median turnaround time in days from sample collection to dispatching VL test results, and iii) the proportion with viral suppression among those who underwent VL testing and factors associated with not achieving viral suppression.

## METHODS

This was a cohort study using programmatic data routinely collected in the ART centres of Sierra Leone. Sierra Leone has an estimated population of eight million people, and is characterised by a youthful demographic profile, with a median age of 19.7 years.^17^ Administratively, the country is divided into four provinces and the Western Area, encompassing a total of 16 districts and 190 chiefdoms. The public health care system of the country provides services through 6 tertiary hospitals, 4 regional hospitals, 8 district hospitals, 11 secondary hospitals, and peripheral health units in chiefdoms.^18^

HIV care is coordinated by the national AIDS control programme (NACP), with designated staff at ART centres overseeing clinical management. While HIV testing services are provided across all hospital departments, HIV care and treatment is provided free of cost at the ART centres co-located in the hospitals.^13^ Upon diagnosis, children and adolescents are registered in the ART registers and provided with client cards with unique identifiers. Then children and adolescents are initiated on ART with either DTG-based regimen (abacavir/tenofovir + lamivudine + DTG) or non-DTG-based (abacavir/azidothymidine + lamivudine + lopinavir/ritonavir) regimen. The children and adolescents along with the caregivers collect drug refill once every 28 days.^13^

The first VL test is supposed to be done after 6 months of ART initiation, followed by a second VL test at 12 months if the viral suppression is achieved, and subsequently at every 6-month intervals.^13^ VL sample collection usually coincides with medication refills but sometimes caregivers collect ART drugs for the children, requiring separate visit by the children for VL testing. Once the sample is collected for VL testing at the ART centre, it is sent to one of the two central molecular laboratories responsible for VL testing: the Ola During Children’s Hospital in the Western Area Urban and the Makeni Regional Hospital laboratory in Bombali district.^16^ Laboratory results are communicated back to the health facilities using the laboratory information management system (LIMS) portal and are documented in VL registers, ART registers, and client cards.

Upon initial VL testing, patients with undetectable VL (≤50 copies/mL) continue the same ART regimen. Those with moderate viraemia (50–1,000 copies/mL) receive adherence counselling and retesting after 3 months, and those achieving undetectable VL continue the same regimen, whereas those with persistent moderate viraemia continue the same drugs with ongoing support and monitoring every 3 months. Those with VL above 1,000 copies/mL (not achieving viral suppression) are switched to a different ART regimen. All the outcomes are updated in both VL registers and client cards.

### Study population

All children and adolescents registered for HIV care in 31 ART centres of eight high-HIV-burden districts (Bo, Bombali, Kailahun, Kenema, Moyamba, Port Loko, Western Area Rural, and Western Area Urban) after January 2023 were eligible for study. However, only those currently on ART care (visited an ART centre at least once in the last 3 months) during data collection in April to October 2025 were included, and those who were lost to follow-up and died were excluded.

### Data variables and data sources

From April to October 2025, the trained NACP staff prepared a line list of children and adolescents currently on care by extracting data from the ART registers and client cards maintained in the ART centres. The demographic details like age, sex, district, ART centre name, supporting partner for ART centre, and date of ART initiation were extracted. Baseline clinical details like CD4 count, ART regimen, WHO clinical stage, and TB co-infection were extracted. Details of VL testing were extracted from client cards and VL registers: the VL test status (done/not done), number of VL tests done since January 2023, date of last VL test, and last VL test result (suppressed [<1,000 copies/mL]/not suppressed [≥1,000 copies/mL]). The extracted data were entered into Epicollect5, a mobile-based data capture tool.

### Data analysis

The data captured in the Epicollect5 were exported and analysed using Stata 16.1 (College Station, TX, USA: StataCorp LLC). The demographic and clinical details of the study participants were summarised using frequency and percentage. Frequency and percentage with 95% confidence interval (CI) were used to describe VL test status and viral suppression (<1,000 copies/mL) among children and adolescents. The median and inter-quartile range was calculated for VL turnaround time, and it was compared across the study districts and the type of partner supporting ART centre using the Kruskal–Wallis test. Unadjusted and adjusted relative risks with 95% CIs using univariate and multivariate cluster-adjusted modified Poisson regression were calculated to assess the association of demographic and clinical factors associated with not getting any VL test done after registration and not achieving viral suppression in the last VL test. The variables with *P* value < 0.2 in univariate analysis were included in multivariate regression.

### Ethical statement

This study was conducted according to the principles of the Declaration of Helsinki and approved by the Sierra Leone Ethics and Scientific Review Committee (SLESRC No: 014/02/2025). As this was a retrospective study, the need for obtaining informed consent from participants was waived off.

## RESULTS

Of the 950 children and adolescents living with HIV included in the study, 523 (55%) were aged 15–19 years and 689 (55%) were females. Of the total, 301 (33%) were from the Western Area Urban district, 647 (68%) were availing care from ART centres supported by AIDS Healthcare Foundation (AHF), and 476 (50%) were on ART care for >18 months. In total, 664 (70%) were in WHO Stage I and 605 (64%) were initiated on a DTG-based regimen (Table 1).

**TABLE 1.** Socio-demographic and clinical characteristics of children and adolescent registered for ART care in eight districts of Sierra Leone after January 2023 and currently on care (April to October 2025).

Variables	n (%)^A^
Total	950 (100)
Age at registration	
Less than 1	33 (4)
1–4	211 (22)
5–9	111 (12)
10–14	72 (8)
15–19	523 (55)
Sex	
Male	261 (27)
Female	689 (73)
Districts	
Bo	116 (12)
Bombali	49 (5)
Kailahun	50 (5)
Kenema	87 (9)
Moyamba	104 (11)
Port Loko	28 (3)
Western Area Rural	206 (22)
Western Area Urban	310 (33)
ART centre managed by	
AHF	647 (68)
Jheipego	273 (29)
Others	30 (3)
Duration on ART (in months)	
Less than 6	19 (2)
7–12	177 (19)
13–18	278 (29)
>18	476 (50)
WHO staging at ART initiation	
I	664 (70)
II	182 (19)
III and IV	92 (10)
Not recorded	12 (1)
ART regimen	
DTG based	605 (64)
Non-DTG based	345 (36)
Baseline CD4 count	
Less than 200	47 (5)
Equal or more than 200	133 (14)
Not recorded	770 (81)
TB co-infection	
Yes	48 (5)
No	902 (95)

DTG = dolutegravir; ART = anti-retroviral treatment; AHF = AIDS Healthcare Foundation.

A Column percentage.

At least one VL testing (coverage) was conducted in 286 (30%, 95% CI: 27%–33%) children and adolescents currently on ART care. Among children and adolescents who were on care for 12–18 months and >18 months, more than two VL tests were conducted in 5% and 4%, respectively. Those availing care in Western Area Urban district (adjusted relative risk [aRR]: 1.8, 95% CI: 1.0–3.1) and those receiving care from ART centres supported by AHF (aRR: 1.9, 95% CI: 1.4–2.6) and other partners (aRR: 2.2, 95% CI: 1.6–2.9) were at higher risk of not undergoing VL testing (Table 2).

**TABLE 2.** Association of socio-demographic and clinical characteristics with not undergoing VL testing among children and adolescents registered for ART in eight districts of Sierra Leone after January 2023 and currently on care (April to October 2025).

Variables	n	Not undergone VL testing	Unadjusted	Adjusted
		n (%)^A^	RR (95% CI)	RR (95% CI)^B^
Total	950	664 (70)		
Age at registration				
Less than 1	33	20 (61)	1.0 (0.7–1.5)	1.0 (0.6–1.5)
1–4	211	167 (79)	1.3 (1.0–1.8)	1.2 (0.9–1.7)
5–9	111	80 (72)	1.2 (0.9–1.5)	1.1 (0.9–1.4)
10–14	72	44 (61)	1	1
15–19	523	353 (68)	1.1 (0.8–1.6)	1.1 (0.7–1.6)
Sex				
Male	261	192 (74)	1.1 (0.9–1.3)	1.0 (0.9–1.1)
Female	689	472 (69)	1	1
Districts				
Bo	116	93 (80)	2.8 (1.5–5.2)	1.6 (0.9–2.9)
Bombali	49	41 (84)	2.9 (1.5–5.6)	1.5 (0.8–2.9)
Kailahun	50	38 (76)	2.7 (1.4–4.9)	1.5 (0.9–2.7)
Kenema	87	72 (83)	2.9 (1.6–5.2)	1.7 (0.9–2.9)
Moyamba	104	82 (79)	2.8 (1.5–5.0)	1.6 (0.9–2.8)
Port Loko	28	8 (29)	1	
Western Area Rural	206	127 (62)	2.2 (1.1–4.4)	1.4 (0.8–2.4)
Western Area Urban	310	203 (65)	2.3 (1.2–4.3)	1.8 (1.0–3.1)
ART centre managed by				
AHF	647	514 (79)	1.8 (1.3–2.5)	1.9 (1.4–2.6)
Jheipego	273	121 (44)	1	1
Others	30	29 (97)	2.2 (1.6–3.1)	2.2 (1.6–2.9)
Duration on ART (in months)				
Less than 6	19	15 (79)	1.3 (0.9–1.7)	1.1 (0.9–1.4)
7–12	177	141 (80)	1.3 (1.1–1.5)	1.2 (1.0–1.4)
13–18	278	175 (63)	1	1
>18	476	333 (70)	1.1 (0.9–1.3)	1.0 (0.9–1.2)
WHO staging at ART initiation				
I	664	464 (70)	1	1
II	182	21 (66)	1.0 (0.8–1.1)	1.1 (0.9–2.3)
III and IV	92	71 (77)	1.1 (0.9–1.3)	1.0 (0.8–1.2)
Not recorded	12	8 (67)	1.0 (0.6–1.5)	1.3 (0.7–2.4)
ART regimen				
DTG based	605	408 (67)	1	
Non-DTG based	345	256 (74)	1.1 (0.9–1.3)	
Baseline CD4 count				
Less than 200	47	30 (64)	1	
Equal or more than 200	133	88 (66)	1.0 (0.8–1.4)	
Not recorded	770	546 (71)	1.1 (0.8–1.5)	
TB co-infection				
Yes	48	38 (79)	1.1 (1.0–1.3)	1.0 (0.8–2.2)
No	902	626 (69)	1	

ART = anti-retroviral treatment; AHF = AIDS Healthcare Foundation; VL = viral load; RR = relative risk; CI = confidence interval.

A Row percentage.

B Generalised linear model using modified Poisson regression.

The median (IQR) turnaround time from sample collection for the VL test to the dispatch of results was 36 days (17–72). The turnaround time varied significantly by district (*P* < 0.001) from 6 days (IQR: 3–15) in Bombali to 65 days (IQR: 19–98) in Kenema (Table 3).

**TABLE 3.** Median duration of days between sample collection and dispatch of VL results among children and adolescents registered after January 2023 in ART centres of eight districts of Sierra Leone, stratified by districts and partner managing ART centre.

Variables	Underwent VL testing	Duration from sample collection to dispatch of VL results	*P* Value^A^
		Median (IQR)	
Total	286	36 (17–72)	
Districts			
Bo	23	61 (52–90)	<0.001
Bombali	8	6 (3–15)	
Kailahun	12	25 (3–69)	
Kenema	15	65 (19–98)	
Moyamba	22	29 (21–53)	
Port Loko	20	27 (11–52)	
Western Area Rural	79	29 (18–53)	
Western Area Urban	107	53 (15–82)	
ART centre managed by			
AHF	133	35 (17–680)	0.1454
Jheipego	152	40 (23–72)	
Others	1	—	

VL = viral load; ART = anti-retroviral treatment; AHF = AIDS Healthcare Foundation; IQR = inter-quartile range.

A Kruskal–Wallis test.

Viral suppression was achieved in 247/286 (86%, 95% CI = 82%–90%) children and adolescents who underwent VL testing. Those aged 1–4 years (aRR: 3.8, 95% CI: 1.7–8.5), those on ART for <6 months (aRR: 3.4, 95% CI: 1.5–7.9), and those in AHF-supported facilities (aRR: 2.2, 95% CI: 1.2–3.9) had a higher risk of not achieving viral suppression (Table 4).

**TABLE 4.** Association of socio-demographic and clinical characteristics with viral load suppression among children and adolescents registered for ART in eight districts of Sierra Leone after January 2023 and currently on care (April to October 2025).

Variables	n	Viral suppression not achieved^A^	Unadjusted	Adjusted
		n (%)	RR (95% CI)	RR (95% CI)
Total	286	39 (14)		
Age at registration^B^				
Less than 1	13	6 (46)	7.1 (3.2–15.8)	5.1 (1.0–27.5)
1–4	44	10 (23)	3.5 (1.6–7.5)	2.5 (1.1–6.0)
5–9	31	8 (26)	4.0 (1.5–10.3)	2.4 (0.9–6.5)
10–14	28	4 (14)	2.2 (0.8–6.1)	1.3 (0.4–4.8)
15–19	170	11 (6)	1	1
Sex				
Male	69	16 (23)	2.2 (1.3–3.7)	1.1 (0.8–1.6)
Female	217	23 (11)	1	1
Districts				
Bo	23	8 (35)	5.3 (1.6–18.1)	3.2 (1.3–8.0)
Bombali	8	3 (38)	5.7 (2.1–15.9)	2.9 (1.1–7.4)
Kailahun	12	3 (25)	3.8 (1.5–9.7)	1.3 (0.5–3.0)
Kenema	15	5 (33)	5.1 (2.1–12.6)	3.1 (1.5–6.1)
Moyamba	22	1 (5)	0.7 (0.3–1.7)	0.5 (0.3–1.0)
Port Loko	20	1 (5)	0.8 (0.1–6.7)	1.2 (0.1–13.2)
Western Area Rural	79	11 (14)	2.1 (0.7–6.9)	1.4 (0.7–2.8)
Western Area Urban	107	7 (7)	1	1
ART centre managed by				
AHF	133	30 (23)	3.8 (1.8–8.1)	2.2 (1.2–3.9)
Jheipego	152	9 (6)	1	1
Others	1	0 (0)	—	—
Duration on ART (in months)^C^				
Less than 6	4	3 (75)	6.3 (3.4–11.7)	3.4 (1.5–7.9)
7–12	36	6 (17)	1.4 (0.7–2.8)	1.3 (0.6–2.5)
13–18	103	13 (13)	1.1 (0.5–2.1)	1.3 (0.8–2.2)
>18	143	17 (12)	1	1
WHO staging at ART initiation				
I	200	28 (14)	1.7 (0.7–4.1)	
II	61	5 (8)	1	
III and IV	21	5 (24)	2.9 (0.7–12.5)	
Not recorded	4	1 (25)	2.1 (0.5–20.3)	
ART regimen				
DTG based	197	17 (9)	1	1
Non-DTG based	89	22 (25)	2.9 (1.5–5.4)	0.8 (0.4–1.8)
Baseline CD4 count				
Less than 200	17	4 (24)	2.6 (0.5–13.9)	
Equal or more than 200	45	4 (9)	1	
Not recorded	224	31 (14)	1.6 (0.5–5.2)	
TB co-infection				
Yes	10	2 (20)	1.5 (0.4–6.3)	
No	276	37 (14)	1	

PR = prevalence ratio; RR = relative risk; CI = confidence interval; DTG = dolutegravir; VL = viral load; ART = anti-retroviral treatment; AHF = AIDS Healthcare Foundation.

A Viral load suppression is <1,000 copies/mL.

B Row percentage.

C Generalised linear model using modified Poisson regression.

## DISCUSSION

This cohort study on VL testing coverage and viral suppression among children and adolescents in Sierra Leone has three key findings of programmatic relevance. First, VL testing coverage is low, with fewer than one in three children and adolescents undergoing testing. Second, the VL test turnaround time is long, taking more than 1 month from sample collection to dispatch of results. Finally, the viral suppression rate of 86% is appreciable but well short of the 98% target set in the country.

For the first time in Sierra Leone, this study among children and adolescents has provided insights into VL testing coverage and viral suppression in accordance with global HIV monitoring guidelines and the third UNAIDS 95-95-95 target, respectively.^3–5^ As the study highlights the gaps in HIV care provision among key populations, the findings are relevant to national and global HIV programmes to assess their progress towards ending inequities in HIV care and achieving SDG target.^19,20^

Strengths of the study include the use of routine programme data from multiple districts reflecting the real-world programmatic performance, and a comprehensive assessment of both VL testing coverage and viral suppression. The limitations of the study include potential overestimation of VL testing coverage and viral suppression rates due to the exclusion of children and adolescents who were lost to follow-up or died, a relatively small sample size for assessing factors associated with not achieving viral suppression, and a lack of qualitative exploration to understand the reasons for VL testing gaps. Despite these limitations, the study findings have implications for the national and global HIV programme. First, the VL testing coverage of 30% is alarmingly low, falling well below the global thresholds for routine monitoring (95%).^5^ The low VL testing coverage may be due to VL testing through centralised laboratory system and inconsistent sample transportation. Additionally, differences across the districts and supporting partners might be due to variations in the health care workforce and health care workers’ training. However, with low VL testing coverage, treatment failure among several children and adolescents on HIV care might remain undetected, contributing to increased drug resistance, higher transmission risks, and poorer long-term health outcomes.^9,21^

Similar to our findings, low VL testing coverage was reported from Malawi and Zimbabwe due to logistical issues in implementing centralised VL testing.^6,22^ In contrast, higher VL testing coverage was reported in South Africa (82%–90%) and in Cameroon (60%), with improved access to VL testing with decentralised testing infrastructure and robust supply chain management.^6,23^ Thus, there is need for decentralising VL testing through point-of-care technologies, strengthening partner-specific training on HIV care protocol, and integrating VL sample collection with routine drug refill visits to minimise missed opportunities.^24^

The 36-day turnaround time for VL testing exceeds the WHO-recommended threshold of 28 days.^5^ Bombali district achieved shorter turnaround times (6 days), likely benefiting from proximity to a central laboratory, while distant district like Kenema (65 days) had longer turnaround times. The finding of long turnaround time with geographic variations is consistent with studies from Ethiopia (7 months) and from PEPFAR-supported programmes.^6,25^ Transportation challenges and laboratory backlogs in rural settings are major factors contributing to such long turnaround times.^25^ However, such long turnaround times can potentially delay critical interventions like adherence counselling or regimen optimisation, exacerbating risks of immunological decline and opportunistic infections.^26^ Thus, there is a need to strengthen the newly established integrated hub-and-spoke sample transportation network and leverage digital platforms, such as LIMSs, to enable real-time tracking of VL test results.^27^

Finally, viral suppression rates of 86% among children and adolescents, while encouraging, mask inequities, with <80% suppression in children and those on non-DTG-based regimens. The overall viral suppression rate is consistent with that reported from Ethiopia (82%–85%) and South Africa (70%–84%).^25,28^ Similar to our study, lower viral suppression in younger children was reported from Cameroon and Malawi, often due to caregiver-dependent adherence, formulation challenges, and delayed DTG rollout.^23,28^ The better viral suppression among those on DTG regimens supports evidence from multi-regional studies showing a 20%–30% increase in viral suppression with DTG-based regimens compared with other options.^29,30^ Thus, there is a need to continue expanding the DTG-based regimen and fixed-dose combinations, improve caregiver counselling for achieving better medication adherence, and build the operational capacity of supporting partners.

## CONCLUSION

This cohort study among children and adolescents receiving care at ART centres in eight districts of Sierra Leone showed that one third have undergone VL testing, that it takes >30 days to receive VL test results after sample collection, and that appreciably >85% of those tested have achieved viral suppression. To improve VL testing coverage, the NACP should prioritise training health care workers in HIV care protocols, improve sample collection and transportation, and routinely monitor VL testing coverage and turnaround time.
